# Potential therapeutic misadministration due to inappropriate electron beam field shaping

**DOI:** 10.1120/jacmp.v1i3.2641

**Published:** 2000-06-01

**Authors:** Arthur J. Olch, Robert Fallen, Jill Conrad, Robert S. Lavey

**Affiliations:** ^1^ Radiation Oncology Program Childrens Hospital Los Angeles 4650 Sunset Boulevard Los Angeles California 90027

**Keywords:** electron beam, electron applicator, field shaping, dosimetry error

## Abstract

Lead or cerrobend blocking strips are used to shape electron treatment fields when an appropriate custom insert is not available. For the Varian 2100C accelerator, the structural supports of the electron applicators impede the free placement of these field‐shaping strips on the open custom insert frame while placement at the top of the applicator is unimpeded. We have investigated the dosimetric ramifications of placing field shaping strips at the top level of the 15×15 applicator for 6, 9, and 16 MeV electrons. Our results demonstrate as much as a 30% dose decrease and 2 cm penumbral increase when this is done compared to field shaping at the insert level. The magnitude of this dosimetric error qualifies as a therapeutic misadministration in many states depending on how many treatments are delivered in this manner. Based on this finding, we recommend that routine use of lead strip blocking be discouraged in favor of custom inserts due to the potential for inappropriate placement on some linear accelerators.

PACS number(s): 87.53.–j, 87.52.–g

## INTRODUCTION

Electron beam field shaping is typically accomplished by one of two methods. In the first method, an appropriate thickness of lead can be placed on the patient's skin to define the area to be irradiated. In the second more frequently used method, the linear accelerator has an electron applicator that has at its exit a place for a custom‐made cerrobend insert that then defines the field shape. Either of these field‐shaping methods can produce the desired field shape and intensity. When a custom cerrobend insert is not available and the patient's treatment field is planned on the treatment couch immediately followed by treatment, there is the need to create the field shape from available cerrobend or lead strips. However, for some accelerators, i.e., Varian 1800, Clinac 20, 2500, 2100C, 2300C, and the new EX series high energy machines (Varian Oncology Systems, Palo Alto, CA), the applicator structure can impede the correct placement of these blocking strips at the location of the insert, but provides an open surface at the top of the applicator upon which blocking strips may be placed. Although placing the strips at the top of the applicator may be faster and more convenient than at the insert level, dose delivery errors may result. This procedure has been reported to us as having been performed at multiple radiation therapy facilities in the United States. In this study, we quantify the potential dosimetric errors created by using the top of a Varian 2100C electron applicator as a shelf to place cerrobend strips for field shaping.

## METHODS AND MATERIALS

**Figure 1:**
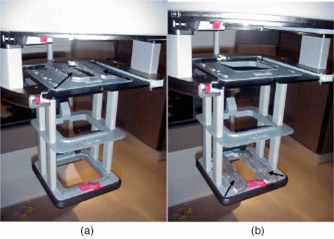
(Color) Varian 2100C 10 cm×10 cm electron applicator. (a) Two cerrobend strips (identified by arrows) can be seen placed on top of the applicator to form a 3‐cm‐wide aperture. (b) Two cerrobend strips (identified by arrows) can be seen placed on the frame at the location of the custom insert to form a 5‐cm‐wide aperture. In both cases, a 5‐cm‐wide field projects to isocenter.

All measurements were performed on a Varian 2100C linear accelerator using the 15 cm×15 cm electron applicator. Measurements of beam profiles were made with Kodak XV‐2 film (Eastman Kodak Co., Rochester, NY) and of $d_{\max}$ dose with a PTW 30001 0.6–cc farmer‐type ionization chamber (PTW New York Corporation, Hicksville, NY) and Keithley 35040 electrometer (Keithley Instruments, Inc., Cleveland, OH). The effects of applying field shaping cerrobend slabs at the top of the applicator (top‐level) [Fig. 1(a)] vs applying these same shaping devices at the level of the cerrobend insert (insert‐level) [Fig. 1(b)] were measured for 6, 9, and 16 MeV electrons.

In each case, a 5‐cm‐wide, 15‐cm‐long light field was obtained at the isocenter. Since the top‐level of the electron applicator is 40 cm from the isocenter, the actual width of the opening produced by the two cerrobend slabs was 3 cm, while at the insert level, which is 5 cm from the isocenter, the width was 4.8 cm.

Relative dose was determined for each field shaping method by using the ionization chamber inserted in a polystyrene phantom. An appropriate buildup thickness of polystyrene was placed on top of the phantom depending on the electron energy under investigation. The top of the phantom was always set to the isocenter and the beam was directed downward on it in the vertical plane. For each energy, measurements were also made for the open field at $d_{\max}$. At least three measurements were taken for each combination of energy and shaping method and were then averaged.

Dose uniformity was tested for the two field shaping configurations by placing a sheet of film on a polystyrene backscattering slab and then placing a polystyrene slab of thickness equal to the $d_{\max}$ depth for each electron energy on top of the film. The slabs were placed on the treatment couch, centered under the beam, so that the surface of the top slab was at the isocenter. In all cases, the gantry was set so that the beam was directed downward in the vertical plane. 40 MU were delivered for each energy for the insert‐level shaping while 57, 49, and 44 MU were delivered for 6‐, 9‐, and 16‐MeV electrons, respectively, for the top‐level shaping in order to produce approximately the same optical density on each film. These monitor units were determined from the relative dose measurements found when using the ionization chamber. A Multidata film scanner (Multidata Systems International Corp., St. Louis, MO) scanned each film through central axis across the 5‐cm dimension and each resulting profile was corrected by using the appropriate optical density to dose conversion function.

## RESULTS

**Figure 2:**
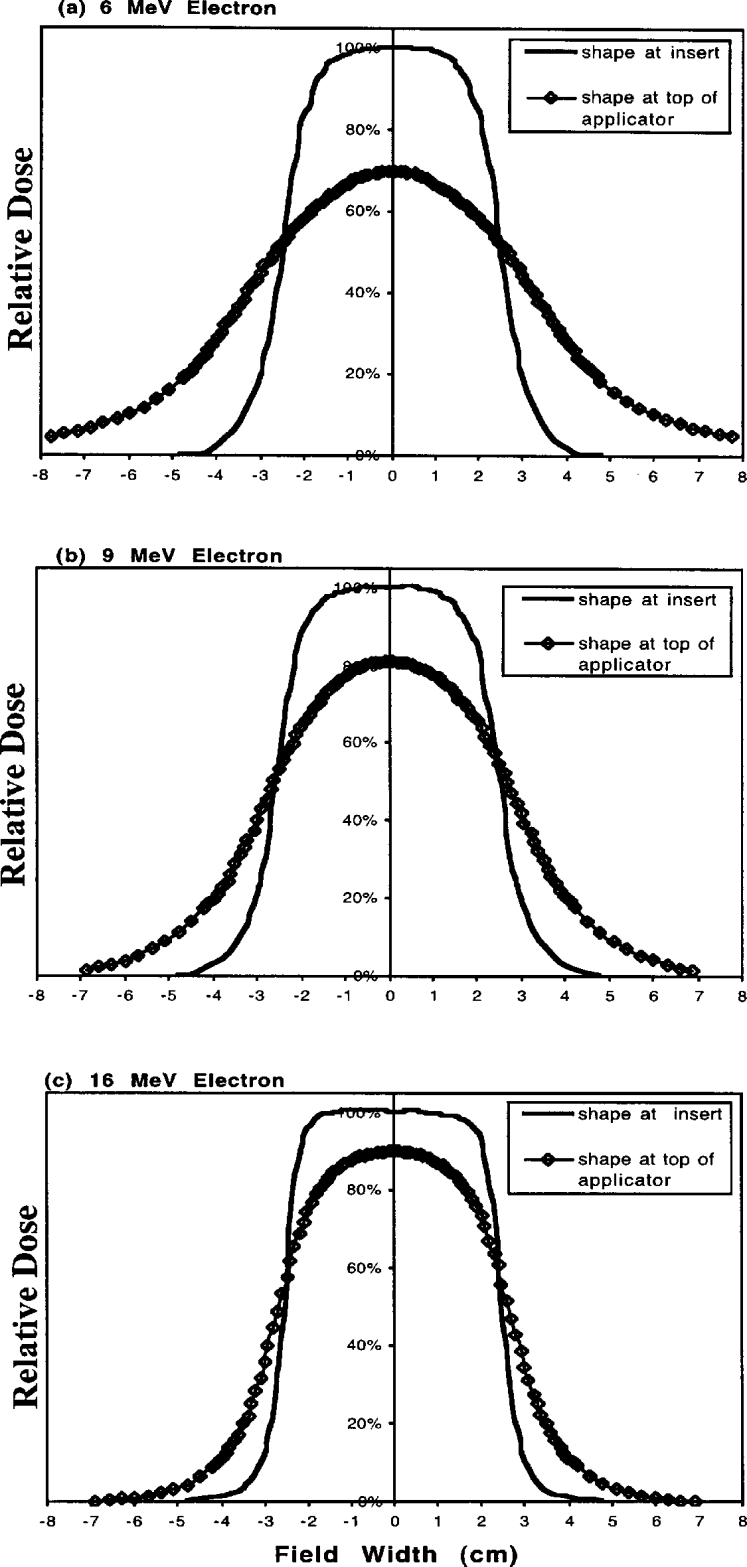
Dose profiles for the (a) 6‐MeV, (b) 9‐MeV, and (c) 16‐MeV electron beams.

For each energy, the insert‐level shaped 15 cm×5 cm fields produced doses within 2% of the expected doses relative to the open 15 cm×15 cm fields. This finding confirms that the method used to create the fields at the insert‐level was equivalent to having used a custom insert. Table I shows the results for the relative dose and profile measurements for both field‐shaping methods, and Fig. 2 displays the dose profiles for the insert‐level shaped fields superimposed on the top‐level shaped fields.

**Table I:** Dose and penumbral differences between top‐level and insert‐level field shaping.

		Top‐level blocking		Insert‐level blocking	
Energy (MeV)	% Dose decrease for top‐level shaping vs expected	FWHM (cm)	20–80 % width (cm)	FWHM (cm)	20–80 % width (cm)
6	30	7.2	3.1	5.0	0.9
9	19	6.0	2.3	5.1	0.9
16	10	5.5	1.5	5.1	0.5

In Figs. 2(a)–2(c), the dose is normalized to 100% for each of the insert‐level shaped fields for each energy. The height of the top‐level shaped field profiles show that the dose for top‐level shaping is 30%, 19% and 10% less than the insert‐level shaping for 6, 9, and 16 MeV, respectively. Figure 2 also demonstrates the degree of broadening of the beam profiles for each energy when top‐level shaping is done compared to insert‐level shaping. The insert‐level shaping provided beam profiles with full width at half maximum (FWHM) of very close to 5 cm. For the top‐level shaped profiles, although the FWHM of the intended dose (central axis dose of the insert‐level shaped field) is 5.4 cm for each energy, the FWHM of each of these profiles was 7.2, 6.0, and 5.5 cm for 6, 9, and 16 MeV, respectively. The 20–80% penumbral width on each side of the beam for 6, 9, and 16 MeV was 3.1, 2.3, and 1.5 cm for top‐level shaping and 0.9, 0.9, and 0.5 cm for insert‐level shaping, respectively.

## DISCUSSION

In a busy department where electron beam treatments are frequently planned in the treatment room and then immediately delivered, custom made shaping for the first one or more treatments is often not practical. If a previously made insert is not suitable, lead strips are often used to define the field shape by placing them in the electron applicator so that they produce a light field shadow which matches the lines drawn on the patient by the radiation oncologist. However, due to the construction of the applicator, it may be difficult to place these lead strips on the area surrounding the cerrobend insert [Fig. 1(b)]. For the Varian 2100C, one has difficulty placing the lead strips at the insert level due to the presence of supporting bars. At some treatment facilities, the lead strips are therefore routinely placed on top of the applicator (between the top surface and the end of the collimator) where there are no impediments to placement [Fig. 1(a)]. In either location, one can position the lead strips to shadow the prescribed area of the patient. For other linear accelerators, the ability to place lead strips at different locations in the applicator depends on the particular design and was not investigated for this report.

It is well known that the distance from the electron beam‐shaping device to the patient surface is a critical parameter in the determination of both the absolute dose as well as the shape of the beam.^1^ ^–^ ^3^ As the beam passes through the electron applicator, a cohort of electrons is traveling along rays that emanate from the virtual source, and a cohort is traveling at various scattered angles due to collisions with the jaw faces, the inside of the applicator, as well as with the edges of the field shaping insert.^3^ ^–^ ^8^ The distance the scattered electrons travel laterally will be greater than that of the divergent electrons after passing through the shape‐defining aperture, especially if there is a large distance between aperture and skin surface. In our study, the top‐level defined beam (confined now only by the 15×15 standard insert) travels about 40 cm to isocenter vs 5 cm for insert‐level shaping. In addition, when field shaping is done at the top level, the aperture for electrons to pass through is only 3‐cm wide in order to produce the 5‐cm light field width at the isocenter. Also, consider scattered electrons that are outside the divergent rays connecting the edges of the 3‐cm‐wide aperture at the top level and the 5‐cm‐wide aperture at the insert level. Some of these would have scattered into the field by the time they reached the insert level but are instead stopped in the top‐level shaping material and fail to contribute to the dose at central axis. Thus the 3‐cm‐wide top‐level opening is at the same time stopping some scattered electrons from reaching the isocenter as well as permitting electrons, otherwise blocked by the insert‐level cerrobend, from reaching several centimeters lateral to the intended field border. These effects are largest for the smallest electron energy. In addition, inspection of Fig. 2 suggests that the area under the insert‐level shaping curves is about equal to the area under the top‐level shaping curves. Apparently, for top‐level shaping, the dose reduction at central axis is about equal to the dose increase outside the intended field. For the 6‐MeV energy, the beam was broadened beyond the 50% dose level such that the penumbra increased by more than 2 cm on each side of the beam. As expected, the 16‐MeV beam was least affected by top‐level shaping, with a 10% dose decrease and a 0.2 cm and 1.0 cm broadening of the 50% and penumbral dose levels on each side of the beam, respectively. The 9‐MeV beam was affected intermediately between the 6‐ and 16‐MeV beams.

The findings of this study demonstrate that shaping electron fields by applying lead blocking material at a position closer to the source of radiation decreases the delivered dose by up to 30% for low energies as well as broadens the beam to extend well past the light field border. These dosimetry errors could significantly contribute to lack of tumor control as well as cause radiation toxicity to sensitive normal structures thought to be outside the radiation field.

It is our suggestion, based on these findings, that routinely shaping electron beams with lead strips should generally be avoided. Although properly placing these strips on the insert level of the electron applicator produces beam characteristics consistent with those produced when using a custom insert, the temptation to place the lead strips in inappropriate but more convenient places in the applicator risks misadministration of the treatment. If field‐shaping strips are to be used for emergency treatments, the staff should be trained to ensure that the strips are properly placed at the insert level.
